# Glucosamine-magnesium composite as functional carriers: structural characteristic, controlled Mg^2+^ release and emulsion stability

**DOI:** 10.3389/fnut.2025.1651560

**Published:** 2025-09-03

**Authors:** Yayuan Zhai, Yue Chen, Ang Gao, Yichen Dou, Yang Gao, Heran Xie

**Affiliations:** ^1^College of Public Health and Health Sciences, Tianjin University of Traditional Chinese Medicine, Tianjin, China; ^2^National Institute of Sports Medicine, Beijing, China

**Keywords:** Pickering emulsion, D-Glucosamine, stimulus responsiveness, Mg^2+^, emulsion stability, controlled release

## Abstract

This study aimed to develop a glucosamine-magnesium composite (GlcN-Mg) as a novel Pickering emulsion stabilizer for the preparation of GlcN-Mg stabilized Pickering emulsion (GlcN-Mg PE) and systematically characterize its structural properties and emulsification performance. Structural analysis revealed that Mg^2+^ coordination reduced GlcN⋅HCl particle size from 1,117 ± 222.58 to 393.8 ± 45.42 nm, expanded its crystal lattice, and created a porous structure with a 22.9 ± 1.80 nm pore size. *In vitro* studies have shown that GlcN-Mg exhibits exceptional stability in food matrices and controlled Mg^2+^ release during gastrointestinal digestion. Then GlcN-Mg composite was employed to prepare water-in-oil (W/O) GlcN-Mg PE under different homogenization speeds (5,000–25,000 rpm), GlcN-Mg concentrations (0.3%–1.3%), and oil-to-water ratios (3:7–8:2). Rheological analysis indicated that GlcN-Mg PE exhibited a distinct threshold effect under varying conditions, while environmental factors significantly influenced emulsion stability. Furthermore, during *in vitro* gastrointestinal digestion, GlcN-Mg PE exhibited controlled-release ability, with the Mg^2+^ release rate reaching 80.42 ± 1.94% during intestinal digestion. Its stability across a wide range of conditions highlights its potential applications in complex emulsion systems.

## Graphical Abstract

Schematic illustrating the preparation process and experimental operation of glucosamine-magnesium composite (GlcN-Mg) and glucosamine-magnesium composite stabilized Pickering emulsion (GlcN-Mg PE). 
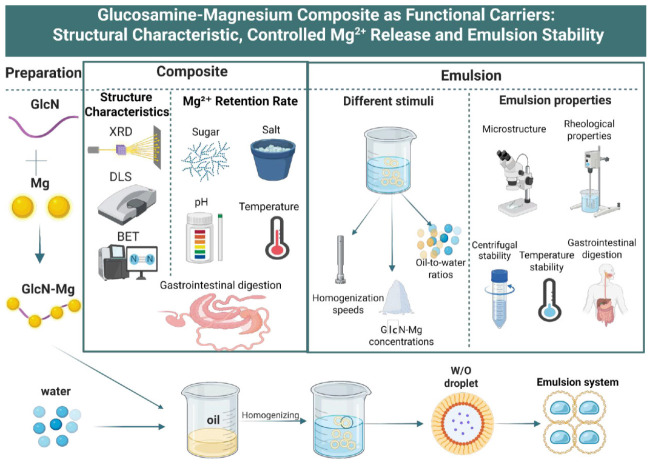

## 1 Introduction

Pickering emulsions, stabilized by solid particles rather than surfactants, were first described by Ramsden (protein assembly at air-water interfaces) (1, 2) and later defined by Pickering as systems utilizing colloidal particles at the oil-water interface (3). Their stability arises from particle adsorption at the interface, which forms a barrier against droplet aggregation (4). If unadsorbed colloidal particles are present in a continuous phase, they may create a three-dimensional network structure that restricts droplet movement in the dispersed phase, thereby improving the stability of the emulsion (5). These emulsions are widely applied in drug delivery [e.g., Li’s self-healing pectin/chitosan hydrogels for controlled release (6)], food technology [e.g., Wang’s gelatin-stabilized O/W emulsions for intelligent packaging (7)], and cosmetics [e.g., improved texture and active ingredient penetration (8–11)]. Current research on Pickering emulsions has primarily focused on exploring food-grade particles, particularly proteins and polysaccharides. Despite progress, studies on particle emulsifiers remain limited, and there is an urgent demand for green, safe solid particles to stabilize Pickering emulsions. In addition to the intrinsic properties of the particles, key factors such as particle concentration, oil-to-water ratio, and homogenization conditions significantly influence emulsion stability. Therefore, investigating the effects of these parameters is critical.

D-Glucosamine (GlcN), a derivative of D-glucose with an amino group substituting the hydroxyl group at the C2 position (12), plays critical roles in maintaining articular cartilage health by stimulating chondrocyte-mediated proteoglycan synthesis. This process preserves extracellular matrix integrity and alleviates osteoarthritis symptoms (13). As a precursor for glycoproteins and glycolipids, GlcN also facilitates cellular recognition, communication, and signal transduction (14, 15). Additionally, GlcN forms water-soluble metal coordination composites through its amino and hydroxyl groups, enabling broad applications in pharmaceuticals, food, and functional materials. The coordination between GlcN with Zn^2+^ exhibits specific structures and functional properties and is of great interest in biochemical systems and drug development (16–18). The coordination with Cu^2+^ and other metals has also been applied (19), but the coordination of GlcN with Mg^2+^ and its associated conformational relationship have not been systematically reported.

Mg^2+^ is an essential element for the human body and participates in more than 300 enzymatic reactions, for example, as an activator of enzymes involved in sugar metabolism, fat metabolism, protein synthesis, and nerve impulses (20). In the cardiovascular system, Mg^2+^ can regulate heart rhythm, stabilize blood pressure and maintain the normal diastolic function of blood vessels by affecting vascular smooth muscle cells. Additionally, Mg^2+^ is a constituent of bone and is important for bone health and metabolism (21, 22). While the physiological importance of Mg^2+^ is well-established, its efficient delivery and stabilization in functional systems like emulsions remains a challenge. Unlike typical Pickering emulsifiers, GlcN-Mg is water-soluble yet retains emulsion-stabilizing capacity. This intriguing property may arise from adsorption of soluble particles at the oil-water interface. Similar behavior has been documented for water-soluble chitosan-Zn^2+^ complexes, which stabilize emulsions via interfacial adsorption even with their solubility (23); this supports the plausibility of such a mechanism for GlcN-Mg.

In this study, a GlcN-Mg composite was employed as an emulsifier to prepare GlcN-Mg-stabilized Pickering emulsions (GlcN-Mg PE). The effects of three processing parameters—homogenization speed, GlcN-Mg concentration, and oil-to-water (O/W) ratio—on emulsion properties were systematically investigated. Structural analyses (XRD, DLS, BET) and *in vitro* simulated digestion experiments were investigated to elucidate structure-property relationships. This study aims to develop a GlcN-Mg PE, establishing a material foundation for its potential as a controlled Mg^2+^ delivery system with enhanced stability. Such systems exhibit potential in intelligent drug delivery or functional food applications where precise controlled release is required.

## 2 Materials and methods

### 2.1 Chemicals

D-(+)-Glucosamine hydrochloride (GlcN⋅HCl, > 98% purity; Shanghai Macklin Biochemical Co., Ltd., China); Anhydrous magnesium sulfate (MgSO_4_, AR grade; Sinopharm Chemical Reagent Co., Ltd., China); Sodium hydroxide (NaOH, ≥ 96%; Xilong Scientific Co., Ltd., China); Absolute ethanol (C_2_H_5_OH, ≥ 99.7%; Tianjin Fuyu Fine Chemical Co., Ltd., China); Acetone (CH_3_COCH_3_, ≥ 99.5%; Tianjin Damao Chemical Reagent Factory, China); Anhydrous methanol (CH_3_OH, ≥ 99.5%; Aladdin Biochemical Technology Co., Ltd., China); Non-GMO primary soybean oil [COFCO Jiayue (Tianjin) Co. Ltd., China]; Pepsin (from porcine gastric mucosa, ≥ 250 U/mg; Sigma-Aldrich, United States); Bile salt (porcine, > 95%; Solarbio Science and Technology Co., Ltd., China); Pancreatin (from porcine pancreas, 4 × USP; Yuanye Bio-Technology Co., Ltd., China). High-speed shear dispersing homogenizer (FJ200-SH, Shanghai Huxi Industrial Co., Ltd., China), High-speed freezing centrifuge (5804R, Eppendorf, Germany), Rotational viscometer (NDJ-1B, Shanghai Changji Geological Instrument Co., Ltd., China), Optical microscope (YS100, Nikon, Japan), X-ray diffractometer (Rigaku Ultima IV, Rigaku Corporation, Japan), Nano particle size and Zeta potential analyzer (Malvern Zetasizer Nano ZS90, Malvern Panalytical Ltd., United Kingdom), Automatic specific surface area analyzer (ASAP 2460, Micromeritics Instrument Corporation, United States).

### 2.2 Preparation of GlcN-Mg composite

A total of 1.0 g of GlcN⋅HCl and 0.5582 g of anhydrous MgSO_4_ were weighed and added to 20 mL of distilled water. After stirring for 1 h at 23 °C, the pH was adjusted to 6.5 with NaOH solution. After the mixture have reacted for 4 h, three times volumes of acetone were added to the mixture. Anhydrous methanol was added to the mixture followed by stirring. The supernatant was discarded after filtration. The filter residue was washed with anhydrous ethanol until dry. The obtained white powder was GlcN-Mg composite.

### 2.3 Determination of structure characteristics of GlcN-Mg

#### 2.3.1 X-ray diffraction (XRD)

The crystal structure of GlcN⋅HCl and GlcN-Mg was analyzed using an X- ray diffractometer (Rigaku Ultima IV diffractometer, Japan). Diffractograms were collected over a scanning angle range (2θ) from 5°to 90°at a scanning rate of 2°/min, with settings of 40 kV and 40 mA.

#### 2.3.2 Dynamic light scattering (DLS)

The hydrodynamic diameter and surface charge characteristics of GlcN⋅HCl and GlcN-Mg were explored using a nano-particle size and zeta potential analyzer (Malvern Zetasizer Nano ZS90, United Kingdom) at a concentration of 1 mg/mL. Three consecutive measurements at 25 °C were performed.

#### 2.3.3 Nitrogen adsorption-desorption isotherm (BET)

The adsorption-desorption isotherms of GlcN⋅HCl and GlcN-Mg were measured with an automatic specific surface area analyzer (ASAP 2460, United States). Nitrogen was used as the adsorption medium. The BET surface area was calculated according to the Brunauer-Emmett-Teller (BET) method, and the pore size was calculated according to the Barrett-Joyner-Halenda (BJH) method.

### 2.4 Surface elemental analysis

The GlcN⋅HCl and GlcN-Mg were evenly spread as a thin layer on the conductive adhesive. Then, the base plate with the sample-attached conductive adhesive was placed into an ion sputtering instrument for gold sputtering treatment. The energy spectrum software was opened, and the acceleration voltage was set at 10.0 kV, with the working distance of 20.0 mm, for the analysis of main surface elements within a depth range of 50–500 nm on the sample surface.

### 2.5 Determination of GlcN-Mg *in vitro* simulated gastrointestinal digestion

A total of 0.3 g of GlcN-Mg was mixed with 3 mL of simulated gastric fluid containing 1 g/L pepsin in 0.1 mol/L HCl, and the pH was adjusted to 2.0 with 0.1 mol/L HCl. The mixture was placed into a dialysis bag (100 Da cutoff), sealed, and immersed in a beaker containing 300 mL of simulated gastric fluid. Digestion was conducted in a 37 °C water bath for 2 h. Every 20 min, 1 mL of the solution was withdrawn from the beaker and replaced with fresh simulated gastric fluid. A total of 0.3 mL of the post-gastric digestion solution in the dialysis bag was transferred to a new dialysis bag, mixed with 3 mL of simulated intestinal fluid, sealed, and immersed in a beaker containing 300 mL of simulated intestinal fluid. Digestion was conducted in a 37 °C water bath for 2 h. Every 20 min, 1 mL of the solution was withdrawn from the beaker (twice per interval) and replaced with fresh simulated intestinal fluid. The Mg^2+^ content in the withdrawn samples was measured to calculate the Mg^2+^ release rate.


$$\mathrm{Release}\,\mathrm{rate}=\left(W_{2}/W_{1}\right)\times 100\%$$

where: W_1_ = Mg^2+^ content inside the dialysis bag pre-digestion;

W_2_ = Mg^2+^ content in the beaker post-digestion.

### 2.6 Mg^2+^ retention rate of GlcN-Mg

#### 2.6.1 Mg^2+^ retention rate of GlcN-Mg in different sugar solutions

A total of 5 mL of GlcN-Mg (100 mg/mL) was placed into a dialysis bag with a molecular weight cutoff of 100 Da. The dialysis bag was immersed in 100 mL of 100 mg/dL solutions of lactose, fructose, glucose, and sucrose, respectively. The samples were incubated in a 37 °C water bath for 90 min, after which the Mg^2+^ retention rate was measured. The pH of the solution was adjusted to 12–14 using NaOH to precipitate Mg(OH)_2_. A Ca^2+^ indicator was added, and the solution was titrated with EDTA to determine Ca^2+^ content. The pH was then adjusted to 8–10, and Eriochrome Black T was used as an indicator to titrate total Ca^2+^ and Mg^2+^ content. Mg^2+^ content was calculated by subtracting Ca^2+^ titration volume from the total titration volume.


RetentionRate=(1-W/2W)1×100%

where: W_1_ = Mg^2+^ content inside the dialysis bag pre-dialysis;

W_2_ = Mg^2+^ content in the beaker post-dialysis.

#### 2.6.2 Mg^2+^ retention rate of GlcN-Mg in different salt ion concentrations

A total of 5 mL of GlcN-Mg (100 mg/mL) was placed into a dialysis bag with a molecular weight cutoff of 100 Da, which was then immersed in 100 mL of NaCl solutions with concentrations (w/v) of 0.5%, 0.7%, 0.9%, 1.1%, and 1.3%, respectively. After incubation in a 37 °C water bath for 90 min, the Mg^2+^ retention rate was measured.

#### 2.6.3 Mg^2+^ retention rate of GlcN-Mg in different pH values

A total of 5 mL of GlcN-Mg (100 mg/mL) was placed into a dialysis bag with a molecular weight cutoff of 100 Da, which was then immersed in 100 mL of phosphate buffer solutions with pH values of 2.5, 5.0, 6.5, 7.0, and 8.0, respectively. After incubation in a 37 °C water bath for 90 min, the Mg^2+^ retention rate was measured.

#### 2.6.4 Mg^2+^ retention rate of GlcN-Mg in different temperature

A total of 5 mL of GlcN-Mg (100 mg/mL) was placed into a dialysis bag with a molecular weight cutoff of 100 Da, which was then immersed in 100 mL of distilled water. The samples were incubated in water baths at 25 °C, 37 °C, 50 °C, 75 °C, and 100 °C for 30 min, followed by measurement of the Mg^2+^ retention rate.

### 2.7 Preparation of GlcN-Mg PE

#### 2.7.1 Preparation of GlcN-Mg PE with different homogenization speeds

The GlcN-Mg composite (0.7% w/v) was dissolved uniformly in deionized water. Following dispersion, 70% (v/v) soybean oil was added to the aqueous phase. The mixture was then homogenized using a homogenizer at different homogenization speeds (5,000, 10,000, 15,000, 20,000, and 25,000 rpm) for 3 min. The GlcN-Mg PE with different homogenization speeds were obtained.

#### 2.7.2 Preparation of GlcN-Mg PE with different GlcN-Mg concentrations

The GlcN-Mg composite (0.3%, 0.5%, 0.7%, 0.9%, 1.1%, and 1.3% w/v) was dissolved uniformly in deionized water. Following dispersion, 70% (v/v) soybean oil was added to the aqueous phase. The mixture was then homogenized using a homogenizer at a homogenization speed of 20,000 rpm for 3 min. The GlcN-Mg PE with different GlcN-Mg concentrations were obtained.

#### 2.7.3 Preparation of GlcN-Mg PE with different oil-to-water ratios

The GlcN-Mg composite (0.7% w/v) was dissolved uniformly in deionized water. Following dispersion, soybean oil was added to the aqueous phase at different oil-to-water ratios (3:7, 4:6, 5:5, 6:4, 7:3, and 8:2). The mixture was then homogenized using a homogenizer at a homogenization speed of 20,000 rpm for 3 min. The GlcN-Mg PE with different oil-to-water ratios were obtained.

### 2.8 Determination of type of GlcN-Mg PE

A total of 1 mL of freshly prepared GlcN-Mg PE was added to 20 mL of ultrapure water and 20 mL of soybean oil, respectively. The mixtures were allowed to stand for 30 s to observe the morphology of the droplets in the aqueous and oil phases. Emulsion types were determined based on droplet distribution patterns: oil-in-water (O/W) emulsions exhibited dispersed droplets in the aqueous phase with oil phase aggregation. Water-in-oil (W/O) systems demonstrated inverse dispersion behavior (24).

### 2.9 Determination of microstructure of GlcN-Mg PE

The GlcN-Mg PE droplet (5 μL) was spread on a slide and covered with a cover glass. Micrographs of GlcN-Mg PE were obtained using a light microscope and observed at 100 × magnification (10 × objective lens and 10 × eye lens).

### 2.10 Determination of rheological properties of GlcN-Mg PE

The viscosity of GlcN-Mg PE was measured continuously for 5 min at 23 °C and 60 rpm using an NDJ-1B rotational viscometer with rotor No. 1. The viscosity values were recorded every 30 s, and the average value was taken to evaluate the emulsion viscosity.

### 2.11 Determination of stability of GlcN-Mg PE

#### 2.11.1 Determination of droplet size and centrifugal stability of GlcN-Mg PE

The average droplet size of GlcN-Mg PE was determined by the arithmetic mean diameter of no fewer than 50 measured droplets. 50 mL of GlcN-Mg PE was centrifuged in a high-speed freezing centrifuge at 3,000 rpm for 5 min at 4 °C. The stability index (SI) was determined by measuring the volume of the emulsion phase and the total volume of all phases. The formula used to calculate the stability index is as follows:


SI=(Ve/Vs)×100%

where: Vs = volume of the whole sample (mL);

Ve = volume of the emulsion phase (mL).

#### 2.11.2 Determination of temperature stability of GlcN-Mg PE

A total of 10 mL of GlcN-Mg PE was incubated at −20 °C for 24 h. Additionally, 10 mL of the same GlcN-Mg PE was incubated at 4 °C, 25 °C, 50 °C, and 80 °C for 1 h. The emulsion was observed to evaluate its temperature stability. The temperature stability of GlcN-Mg PE was expressed in terms of stability index (SI).

### 2.12 Determination of GlcN-Mg PE *in vitro* simulated gastrointestinal digestion

A total of 0.1 g of GlcN-Mg was emulsified and dispersed in 1 mL of simulated gastric juice containing 1 g/L pepsin in 0.1 mol/L HCl. The mixture was placed in a dialysis bag (100 Da MWCO) and digested in 100 mL simulated gastric juice at 37 °C for 2 h. Post-gastric digestion, 1/10 of the solution was transferred to a new dialysis bag, mixed with 1 mL of simulated intestinal fluid (1.2 g bile salt, 0.2 g pancreatin in 100 mL 0.1 mol/L NaHCO_3_), and digested in 100 mL simulated intestinal fluid for another 2 h at 37 °C. Then measured and calculated the release rate of Mg^2+^.

### 2.13 Statistical analysis

All experiments were performed in triplicate, and data are presented as means ± standard deviations. Statistical significance was evaluated using one-way analysis of variance (ANOVA) with Tukey’s post hoc test, with a significance level set at *p* < 0.05. Data analysis and plotting were conducted using Origin 2024.

## 3 Results and discussion

### 3.1 Structure characteristics of GlcN-Mg

#### 3.1.1 XRD

**FIGURE 1:**
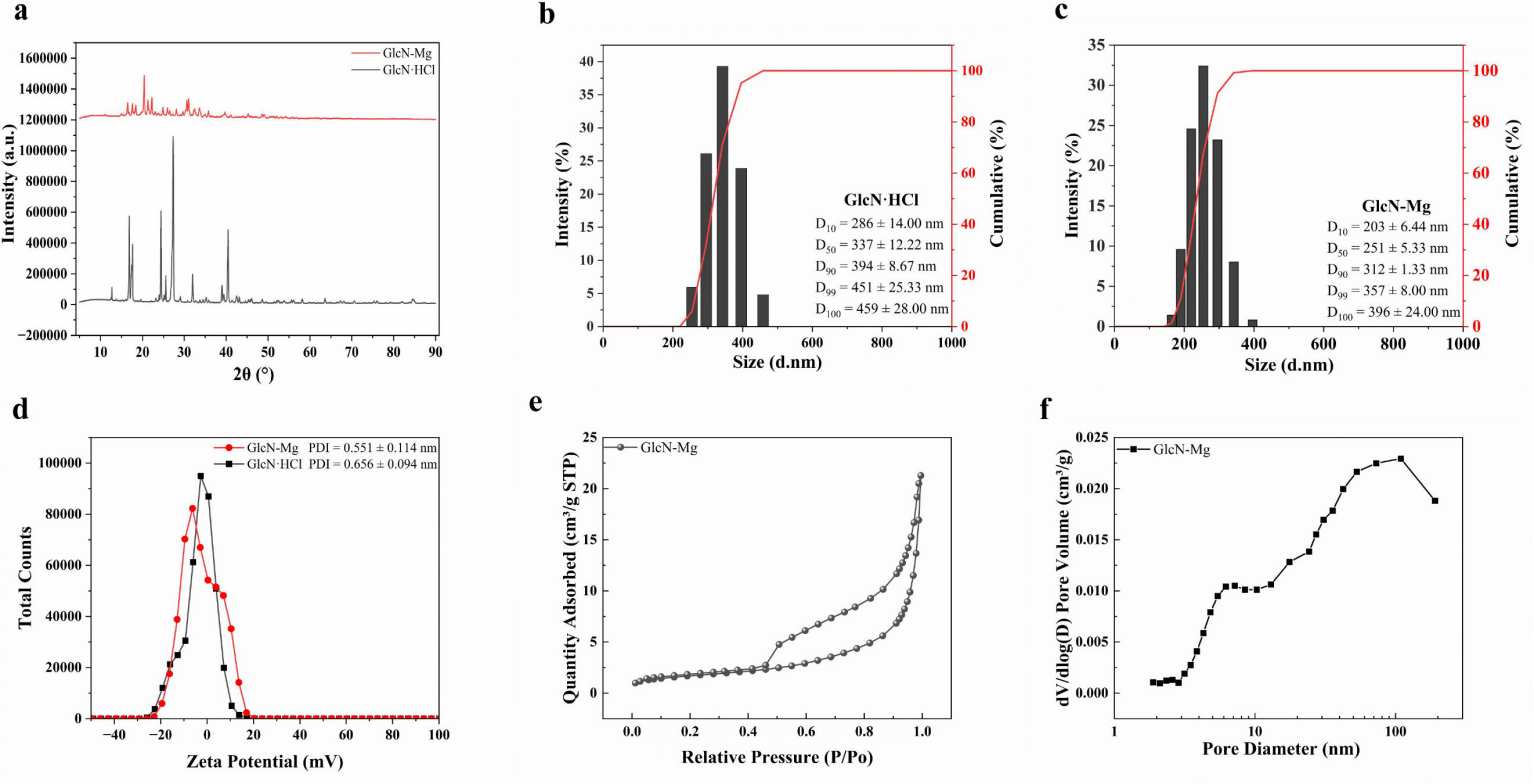
**(a)** X-ray diffraction (XRD) pattern of GlcN⋅HCl and glucosamine-magnesium composite (GlcN-Mg). **(b)** Size distribution and cumulative percentage of GlcN⋅HCl particle. **(c)** Size Distribution and cumulative percentage of GlcN-Mg composite. **(d)** Zeta potential and polydispersity index (PDI) of GlcN⋅HCl and GlcN-Mg. **(e)** Nitrogen adsorption/desorption isotherm of GlcN-Mg. **(f)** Pore size distribution of GlcN-Mg.

The XRD diffraction spectra of GlcN⋅HCl and GlcN-Mg are shown in Figure 1a. The characteristic sharp diffraction peak (2θ = 12.68°) corresponding to the crystalline lattice of GlcN⋅HCl exhibited a marked intensity reduction in GlcN-Mg, decreasing from 77,250 to 30,412 a.u., which reflects partial disruption of the long-range crystalline order (25). GlcN-Mg exhibited crystalline peaks at 16.5°, 24–25° with peak broadening, and low-angle (< 15°) diffuse scattering, suggesting the coexistence of a crystalline phase and an amorphous domain. The systematic peak shifts observed in GlcN-Mg were quantitatively consistent with Bragg’s law, mechanistically attributed to lattice expansion through Mg^2+^ chelation within GlcN⋅HCl (26, 27). These structural signatures demonstrated the formation of a heterogeneous crystalline-amorphous nanoparticle architecture, consistent with structure-property predictions for Mg chelation biopolymer systems (28).

#### 3.1.2 DLS

The changes in fluid dynamic characteristics induced by GlcN-Mg formation are shown in Figures 1b–d. Unmodified GlcN⋅HCl exhibited a Z-average diameter of 1,117 ± 222.58 nm with high polydispersity (PDI = 0.656 ± 0.094), indicative of extensive aggregation driven by intermolecular hydrogen bonding and weak electrostatic repulsion, as reflected by its near-neutral zeta potential (ζ = −3.10 mV). The formation of GlcN-Mg resulted in a reduction of the Z-average diameter to 393.8 ± 45.42 nm with moderate polydispersity (PDI = 0.551 ± 0.114). The observed size refinement and reduced PDI indicate that Mg^2+^ coordination with amino/hydroxyl groups disrupted hydrogen-bonded clusters (29, 30), thereby promoting monodispersity. This coordination disrupted the native hydrogen-bonded clusters and enhanced system stability. The consistently near-neutral zeta potentials suggest kinetic stabilization through coordinative network assembly (19). Compared to GlcN⋅HCl, the smaller particle size of GlcN-Mg composite facilitates effective adsorption at the oil-water interface, likely due to synergistic effects of lower interfacial tension and enhanced amphiphilicity. These properties enable superior interfacial anchoring, as evidenced in prior studies (31, 32).

#### 3.1.3 BET

The nitrogen adsorption/desorption isotherm and pore size distribution of GlcN-Mg are shown in Figures 1e, f. Unmodified GlcN⋅HCl exhibited a Type II isotherm (non-porous characteristics) with monotonic adsorption progression, consistent with previous reports on GlcN⋅HCl (33, 34). GlcN-Mg displayed a Type IV (a) isotherm with an H_2_ (b)-type hysteresis loop (P/P_0_ = 0.45–0.90), indicative of well-developed mesoporosity. The BET surface area increased to 6.1 ± 0.30 m^2^/g, with an average pore diameter of 22.9 ± 1.80 nm (4V/A method) and a pore volume of 0.03 cm^3^/g. Bimodal pore size distributions at 7.2 and 109 nm reflected hierarchical porosity arising from Mg^2+^-induced structural reorganization. The 7.2 nm mesopores originated from interparticle voids between expanded nanocrystalline domains. The 109 nm macropores likely resulted from inter-aggregate spacing of Mg^2+^-coordinated lamellar sheets. This porosity development was attributed to Mg^2+^-mediated disruption of native hydrogen-bonding networks (35, 36).

### 3.2 Surface element analysis

**FIGURE 2:**
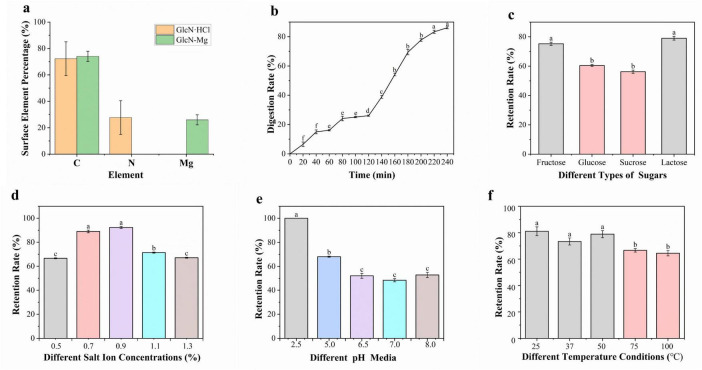
**(a)** The surface element analysis of GlcN⋅HCl and glucosamine-magnesium composite (GlcN-Mg). **(b)** Gastrointestinal digestibility of GlcN-Mg. **(c)** Mg^2+^ retention rate of GlcN-Mg in different sugar solutions. **(d)** Mg^2+^ retention rate of GlcN-Mg in different salt ion concentrations. **(e)** Mg^2+^ retention rate of GlcN-Mg in different pH values. **(f)** Mg^2+^ retention rate of GlcN-Mg in different temperature.

As shown in Figure 2a, after the formation of the composite, surface elemental analysis by energy-dispersive spectroscopy (EDS) revealed a significant decrease in nitrogen content from 27.74 ± 12.83% to 0% on the crystal surface, while the percentage of Mg element increased accordingly. This may be due to the coordination reaction between amino groups and Mg^2+^ to form coordinate bonds with Mg^2+^ ions occupying terminal positions, burying amino groups beneath the carbon/metal matrix.

### 3.3 GlcN-Mg *in vitro* simulated gastrointestinal digestion

The release rate of Mg^2+^ from GlcN-Mg during simulated gastrointestinal digestion was investigated using EDTA titration to explore the dynamic characteristics and patterns of the digestion process. The result is shown in Figure 2b. During the gastric phase, the Mg^2+^ release rate exhibited a gradual yet slower increase. From 0 to 20 min, the release rate rose from 0% to 6.53 ± 1.56%, indicating slow initial Mg^2+^ release. From 20 to 60 min, the release rate increased from 6.53 ± 1.56% to 16.11 ± 0.44%. From 60 to 120 min, the release rate further increased from 16.11 ± 0.44% to 26.11 ± 0.44%, stabilizing toward the end of the phase. The steady increase in Mg^2+^ release during the gastric phase is attributed to the mild and continuous digestive mechanisms of the stomach, primarily driven by gastric acid and pepsin (37), which gradually break down GlcN-Mg, leading to a slow and linear release of Mg^2+^. The stable temperature, pH, and enzyme activity in the gastric environment ensured an orderly digestion process (38, 39). During the transition from gastric to intestinal digestion at 120 min, the Mg^2+^ release rate increased significantly. In the intestinal phase, the Mg^2+^ release rate increased from 26.11 ± 0.44% (end of gastric phase) to a final value of 86.11 ± 0.67%. Key factors influencing Mg^2+^ release include digestive enzymes, pH, and digestion time. Pepsin in the acidic gastric environment initiated the breakdown of GlcN-Mg, while a combination of intestinal enzymes significantly enhanced digestion efficiency in the alkaline intestinal environment (40). The acidic gastric pH favored pepsin activity (41), while the weakly alkaline intestinal pH optimized the activity of multiple digestive enzymes (42). Prolonged exposure to digestive fluids increased the degree of GlcN-Mg breakdown, leading to higher Mg^2+^ release rates over time. Overall, GlcN-Mg exhibited a sustained slow release profile in the gastrointestinal tract, with Mg^2+^ release proceeding gradually through both gastric and intestinal phases rather than undergoing rapid burst release.

### 3.4 Mg^2+^ retention rate of GlcN-Mg

#### 3.4.1 Mg^2+^ retention rate of GlcN-Mg in different sugar solutions

As shown in Figure 2c, GlcN-Mg exhibited distinct Mg^2+^ retention profiles across different sugar solutions. In lactose solutions, GlcN-Mg yielded the highest retention rate, while in sucrose solutions, it showed the lowest. GlcN-Mg retained slightly more Mg^2+^ in lactose compared to other sugars, likely due to lactose’s structure—a disaccharide composed of glucose and galactose linked by a β-(1→4)-glycosidic bond (43). Its relatively large molecular size generates stronger van der Waals forces with GlcN-Mg, stabilizing the complex and reducing Mg^2+^ release. GlcN-Mg exhibits weaker van der Waals interactions with fructose and glucose due to their simpler, smaller structures (44, 45), thereby resulting in intermediate stability levels. GlcN-Mg encounters steric hindrance with sucrose, which is a disaccharide formed by glucose and fructose linked via an α-1,2-glycosidic bond (46); this hinders tight binding and results in the lowest retention rate. Overall, the GlcN-Mg demonstrated good stability in sugar-containing environments.

#### 3.4.2 Mg^2+^ retention rate of GlcN-Mg in different salt ion concentrations

The influence of salt ion concentrations on Mg^2+^ retention rate is shown in Figure 2d. The highest retention rate was observed at a salt ion concentration of 0.9%. where interactions between salt ions and GlcN-Mg were balanced, inhibiting aggregation and degradation. At this concentration, the system maintained optimal stability, with retention rates significantly higher than those at both higher and lower concentrations. Excessively high salt ion concentrations reduced Mg^2+^ retention. This phenomenon may be attributed to excessive binding of high-concentration salt ions to specific groups in GlcN-Mg, which altered the surrounding ionic environment and disrupting intermolecular forces. Additionally, the reduction in tetrahedral water arrangements and shift toward non-tetrahedral structures (47, 48) at a salt ion concentration of 1.3% further disrupt GlcN-Mg’s structure, increasing denaturation and aggregation. Conversely, low salt ion concentrations also led to lower retention rates due to insufficient ionic strength, which failed to provide adequate stabilization, making the GlcN-Mg more susceptible to external influences. Preliminary observations suggested that GlcN-Mg demonstrated broad stability across various physiological environments.

#### 3.4.3 Mg^2+^ retention rate of GlcN-Mg in different pH values

As shown in Figure 2e, GlcN-Mg exhibited pH-dependent stability profiles, with Mg^2+^ retention rates varying non-linearly across different pH conditions. GlcN-Mg demonstrated significantly higher Mg^2+^ retention in acidic environments, with the maximum retention rate observed at pH 2.5, whereas the lowest retention was recorded at neutral pH. This pH-dependent behavior is attributed to specific interactions in acidic conditions: buffer ions interact with GlcN-Mg to stabilize its structure, allowing GlcN-Mg to maintain optimal conformational integrity and preserve active sites critical for Mg^2+^ binding. In contrast, pH 7.0 is near the isoelectric point of GlcN-Mg, where GlcN-Mg exhibits the highest affinity for the surrounding medium. This leads to unfavorable charge distribution and disrupted intermolecular forces, making GlcN-Mg more prone to degradation or aggregation and thus reducing Mg^2+^ retention. Overall, pH variations modulate intramolecular and intermolecular interactions within GlcN-Mg, directly influencing its stability and Mg^2+^ retention capacity.

#### 3.4.4 Mg^2+^ retention rate of GlcN-Mg in different temperature

Figure 2f illustrates the Mg^2+^ retention rate of GlcN-Mg under different temperature conditions. The results showed that as temperature increased from 25 °C to 100 °C, the Mg^2+^ retention rate exhibited a fluctuating but overall stable trend, with no drastic drops observed across the tested range. GlcN-Mg maintained relatively consistent performance under moderate temperatures (25 °C–50 °C), with no significant differences in retention (*P* > 0.05), indicating good adaptability to typical food processing and storage conditions. This thermal stability can be attributed to the molecular interactions within GlcN-Mg: moderate temperature increases may induce reversible conformational adjustments that preserve structural integrity, while excessive heating slightly weakens intermolecular forces but without causing severe disruption. GlcN-Mg demonstrates reasonable thermal stability, supporting its potential application in food-related scenarios with common temperature variations.

### 3.5 Type of GlcN-Mg PE

**FIGURE 3:**
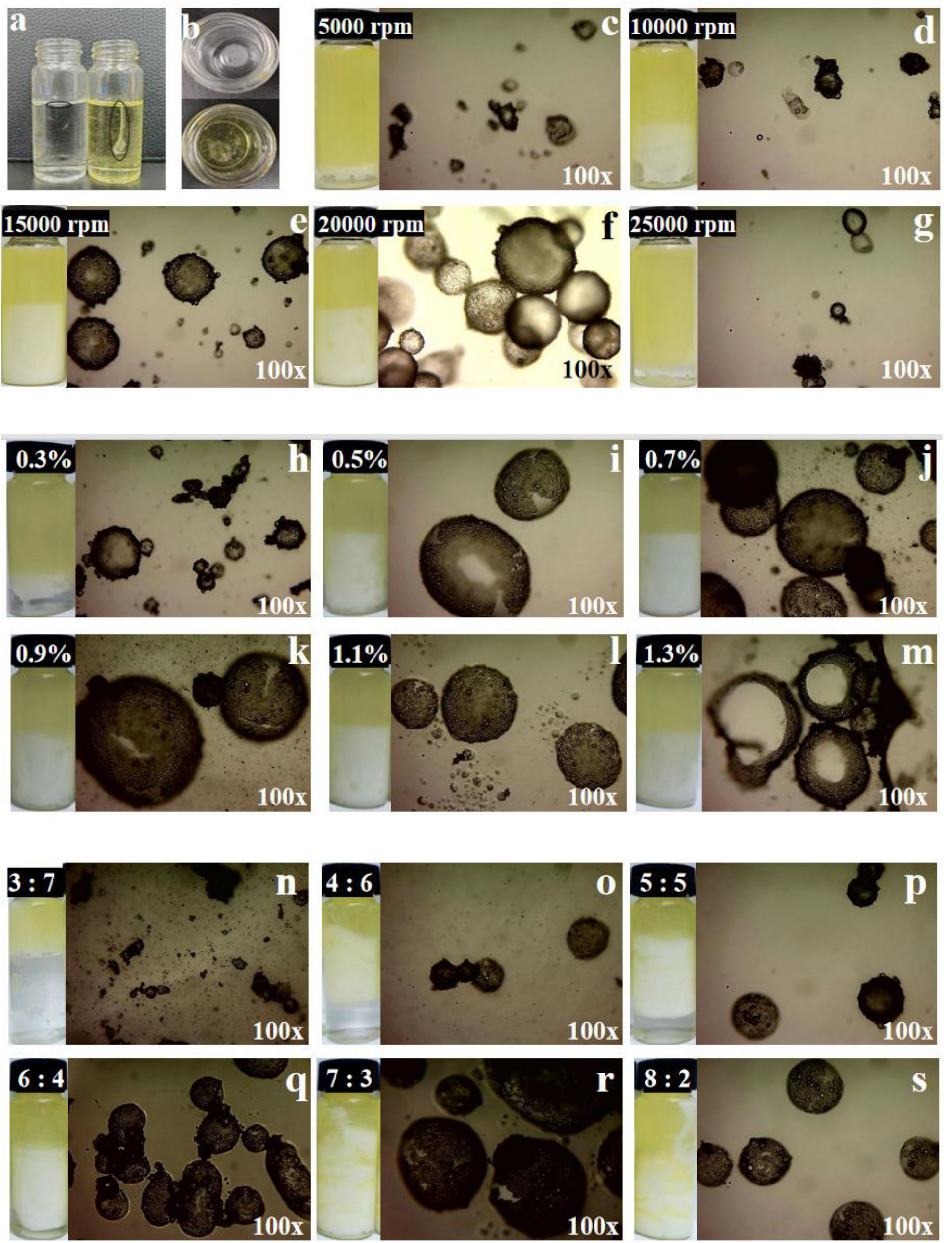
**(a,b)** Photograph of glucosamine-magnesium composite stabilized Pickering emulsion (GlcN-Mg PE) dropped into ultrapure water and soybean oil. **(c–g)** The optical-microscope images of GlcN-Mg PE with different homogenization speeds. **(h–m)** The optical-microscope images of GlcN-Mg PE with different GlcN-Mg concentrations. **(n–s)** The optical-microscope images of GlcN-Mg PE with different oil-to-water ratios.

As shown in Figures 3a, b, the GlcN-Mg PE exhibited distinct dispersion behaviors depending on the continuous phase. In oil phase, the GlcN-Mg PE droplets dispersed continuously, forming a uniform interfacial film. In aqueous phase, the GlcN-Mg PE droplets present discrete spherical droplets formed without coalescence. These phase-dependent morphological characteristics confirm the water-in-oil (W/O) nature of the GlcN-Mg stabilized emulsion.

### 3.6 Microstructure of GlcN-Mg PE

The optical microscope images of GlcN-Mg PE were displayed in Figures 3c–s. The GlcN-Mg composite demonstrated interfacial adsorption ability at the oil-water interface, enabling effective Pickering emulsion stabilization. As droplet uniformity critically determines emulsion functionality (49), systematic analysis of three governing factors: homogenization speed, composite concentration, and oil-to-water ratio is important. Larger and more homogeneous emulsion droplets were observed in GlcN-Mg PE at a moderate homogenization speed. This was because moderate speeds enhanced emulsion property through improved droplet integrity, while extreme shear forces induced structural disruption. The interaction between GlcN and Mg^2+^ improved interfacial activity. GlcN-Mg PE with 0.9% (w/v) GlcN-Mg concentration exhibited the best bridging structure and interconnected networks at the interface. Beyond 0.9% concentration, droplet uniformity plateaued as composite adsorption reached saturation (50). This was because excess composite thickened interfacial layers without modifying droplet dimensions (51). The distribution of emulsion droplets was also influenced by the oil content. Significantly uneven particle size and sporadic distribution were observed when the oil-to-water ratio exceeded 7:3 or was less than 4:6. Specifically, ratios above 7:3 induced phase inversion due to insufficient emulsifier coverage, while ratios below 4:6 led to an excessive water phase, disrupting the W/O structure. Optical microscopy confirmed GlcN-Mg composite localization at droplet interfaces.

### 3.7 Rheological properties of GlcN-Mg PE

Tables 1–3 showed the changes of apparent shear viscosity with increasing shear time. As the shear time increased from 30 to 300 s, the apparent viscosity of all test groups exhibited a decrease in viscosity, attributed to the gradual disintegration of flocculated droplets. This observation indicated that shear-induced breakdown of the intermolecular network and emulsion structure occurred more rapidly than molecular rearrangement. The viscosity of GlcN-Mg PE initially increased with homogenization speed but declined beyond a critical threshold. Moderate homogenization enhanced the composite’s interfacial anchoring and facilitated the formation of a three-dimensional network via controlled droplet expansion. Conversely, excessive speeds disrupted this network via shear-induced structural degradation (52). GlcN-Mg PE with a 1.1% (w/v) concentration had greater viscosity than other concentrations, indicating a stable emulsion system due to the formation of a highly viscous network. Concentrations exceeding 1.1% induced structural defects through composite overcrowding, as evidenced by viscosity deterioration. This emulsifier oversaturation compromised interfacial network integrity through steric hindrance and cooperative binding effects (53). Compared with other oil-to-water ratios, GlcN-Mg PE exhibited the highest viscosity at a 7:3 ratio. Increasing the ratio from 3:7 to 7:3 enhanced viscosity through consolidated droplet packing and intensified interdroplet interactions (54, 55). When the water phase proportion was excessively high, water droplets tended to aggregate, inducing structural imperfections in the GlcN-Mg network. These imperfections impaired the network’s integrity, diminished the system’s flow resistance and consequently lowered viscosity. Conversely, an excessive oil-to-water ratio caused phase-driven interfacial instability, which disrupted structural continuity and reduced viscosity. Rheological analysis indicated that GlcN-Mg PE exhibited a distinct threshold effect under varying conditions. This viscoelastic behavior supports the hypothesis that GlcN-Mg may form a viscoelastic structure at the oil-water interface, contributing to emulsion stability.

**TABLE 1:** Viscosity of glucosamine-magnesium composite stabilized Pickering emulsion (GlcN-Mg PE) with different homogenization speeds.

Homogenization Speeds time (s)	5,000 rpm (mPa⋅s)	10,000 rpm (mPa⋅s)	15000 rpm (mPa⋅s)	20,000 rpm (mPa⋅s)	25,000 rpm (mPa⋅s)
30	27.30 ± 0.01^a^	43.10 ± 0.02^b^	92.60 ± 0.02^c^	177.32 ± 0.02^e^	157.62 ± 0.01^d^
60	26.90 ± 0.01^a^	42.70 ± 0.02^b^	92.40 ± 0.02^c^	177.23 ± 0.02^e^	157.40 ± 0.01^d^
90	26.82 ± 0.01^a^	42.60 ± 0.01^b^	92.30 ± 0.03^c^	176.51 ± 0.02^e^	157.30 ± 0.01^d^
120	26.80 ± 0.01^a^	42.30 ± 0.02^b^	91.63 ± 0.02^c^	176.31 ± 0.03^e^	157.20 ± 0.02^d^
150	26.71 ± 0.01^a^	42.26 ± 0.01^b^	91.53 ± 0.02^c^	175.81 ± 0.02^e^	156.90 ± 0.02^d^
180	26.59 ± 0.02^a^	42.12 ± 0.02^b^	91.34 ± 0.02^c^	175.65 ± 0.03^e^	156.79 ± 0.02^d^
210	26.22 ± 0.02^a^	42.02 ± 0.02^b^	90.51 ± 0.04^c^	175.58 ± 0.02^e^	156.41 ± 0.02^d^
240	26.11 ± 0.01^a^	41.93 ± 0.02^b^	90.32 ± 0.02^c^	175.49 ± 0.02^e^	156.34 ± 0.02^d^
270	25.68 ± 0.02^a^	41.88 ± 0.02^b^	90.22 ± 0.02^c^	175.30 ± 0.02^e^	156.28 ± 0.01^d^
300	25.60 ± 0.02^a^	41.77 ± 0.02^b^	90.04 ± 0.02^c^	175.22 ± 0.02^e^	156.18 ± 0.01^d^
Average viscosity	26.47 ± 0.46^a^	42.27 ± 0.32^b^	91.29 ± 0.81^c^	176.04 ± 0.64^e^	156.84 ± 0.44^d^

All results were shown as mean ± SD. Different superscript letters (a-e) within the same column indicate significant differences (*p* < 0.05) as determined by one-way ANOVA with Tukey’s post hoc test.

**TABLE 2:** Viscosity of glucosamine-magnesium composite stabilized Pickering emulsion (GlcN-Mg PE) with different glucosamine-magnesium composite (GlcN-Mg) concentrations.

GlcN-Mg concentrations time (s)	0.30% (mPa⋅s)	0.50% (mPa⋅s)	0.70% (mPa⋅s)	0.90% (mPa⋅s)	1.10% (mPa⋅s)	1.30% (mPa⋅s)
30	21.60 ± 0.01^a^	98.29 ± 0.02^b^	102.60 ± 0.02^d^	110.59 ± 0.02^e^	113.72 ± 0.02^f^	101.96 ± 0.04^c^
60	21.49 ± 0.01^a^	98.22 ± 0.01^b^	102.40 ± 0.02^d^	110.51 ± 0.01^e^	113.51 ± 0.01^f^	101.89 ± 0.02^c^
90	21.31 ± 0.03^a^	98.17 ± 0.02^b^	102.33 ± 0.02^d^	110.47 ± 0.02^e^	113.40 ± 0.01^f^	101.80 ± 0.01^c^
120	21.21 ± 0.02^a^	97.89 ± 0.02^b^	102.25 ± 0.03^d^	110.42 ± 0.01^e^	113.21 ± 0.02^f^	101.70 ± 0.01^c^
150	21.10 ± 0.01^a^	97.72 ± 0.02^b^	102.09 ± 0.02^d^	110.28 ± 0.02^e^	112.89 ± 0.02^f^	101.60 ± 0.01^c^
180	21.01 ± 0.02^a^	97.61 ± 0.01^b^	101.90 ± 0.01^d^	110.16 ± 0.03^e^	112.80 ± 0.01^f^	101.44 ± 0.03^c^
210	20.90 ± 0.02^a^	97.49 ± 0.02^b^	101.69 ± 0.02^d^	110.03 ± 0.03^e^	112.70 ± 0.01^f^	101.40 ± 0.01^c^
240	20.86 ± 0.01^a^	97.40 ± 0.02^b^	101.52 ± 0.02^d^	109.89 ± 0.02^e^	112.62 ± 0.02^f^	101.20 ± 0.01^c^
270	20.80 ± 0.01^a^	97.29 ± 0.02^b^	101.47 ± 0.02^d^	109.81 ± 0.01^e^	112.58 ± 0.02^f^	101.17 ± 0.02^c^
300	20.70 ± 0.01^a^	97.16 ± 0.02^b^	101.19 ± 0.02^d^	109.54 ± 0.03^e^	112.40 ± 0.01^f^	100.89 ± 0.02^c^
Average viscosity	21.10 ± 0.24^a^	97.73 ± 0.33^b^	101.94 ± 0.39^c^	110.17 ± 0.28^d^	112.98 ± 0.38^e^	101.50 ± 0.28^c^

All results were shown as mean ± SD. Different superscript letters (a-f) within the same column indicate significant differences (*p* < 0.05) as determined by one-way ANOVA with Tukey’s post hoc test.

**TABLE 3:** Viscosity of glucosamine-magnesium composite stabilized Pickering emulsion (GlcN-Mg PE) with different oil-to-water ratios.

Oil-to-water ratios time (s)	3:7 (mPa⋅s)	4:6 (mPa⋅s)	5:5 (mPa⋅s)	6:4 (mPa⋅s)	7:3 (mPa⋅s)	8:2 (mPa⋅s)
30	13.91 ± 0.01^a^	61.27 ± 0.01^b^	108.60 ± 0.02^d^	122.20 ± 0.02^e^	124.92 ± 0.02^f^	96.82 ± 0.02^c^
60	13.84 ± 0.02^a^	61.20 ± 0.02^b^	108.49 ± 0.02^d^	122.10 ± 0.02^e^	124.79 ± 0.02^f^	96.70 ± 0.02^c^
90	13.78 ± 0.02^a^	60.90 ± 0.01^b^	108.39 ± 0.02^d^	121.72 ± 0.02^e^	124.17 ± 0.04^f^	96.20 ± 0.02^c^
120	13.60 ± 0.02^a^	60.50 ± 0.02^b^	108.36 ± 0.01^d^	121.63 ± 0.02^e^	124.07 ± 0.02^f^	95.85 ± 0.02^c^
150	13.40 ± 0.02^a^	60.44 ± 0.03^b^	108.19 ± 0.02^d^	121.51 ± 0.03^e^	123.90 ± 0.01^f^	95.67 ± 0.02^c^
180	13.42 ± 0.02^a^	60.30 ± 0.02^b^	107.89 ± 0.02^d^	121.42 ± 0.01^e^	123.81 ± 0.01^f^	95.32 ± 0.01^c^
210	13.10 ± 0.02^a^	59.90 ± 0.02^b^	107.79 ± 0.02^d^	121.32 ± 0.02^e^	123.76 ± 0.03^f^	95.22 ± 0.01^c^
240	13.02 ± 0.02^a^	59.82 ± 0.01^b^	107.59 ± 0.02^d^	121.22 ± 0.01^e^	123.63 ± 0.03^f^	95.20 ± 0.01^c^
270	12.90 ± 0.01^a^	59.58 ± 0.02^b^	107.42 ± 0.02^d^	120.65 ± 0.03^e^	123.23 ± 0.03^f^	95.06 ± 0.03^c^
300	12.71 ± 0.03^a^	59.40 ± 0.02^b^	107.20 ± 0.02^d^	120.42 ± 0.02^e^	122.86 ± 0.04^f^	94.82 ± 0.02^c^
Average viscosity	13.37 ± 0.35^a^	60.33 ± 0.53^b^	107.99 ± 0.41^d^	121.42 ± 0.41^e^	123.91 ± 0.46^f^	95.69 ± 0.57^c^

All results were analyzed by one-way analysis of variance (ANOVA) with Tukey’s post hoc test, and different letters in the same column indicate significant differences (*p* < 0.05). All results were shown as mean ± SD. Different superscript letters (a-f) within the same column indicate significant differences (*p* < 0.05) as determined by one-way ANOVA with Tukey’s post hoc test.

### 3.8 Stability of GlcN-Mg PE

#### 3.8.1 Droplet size and centrifugal stability

**FIGURE 4:**
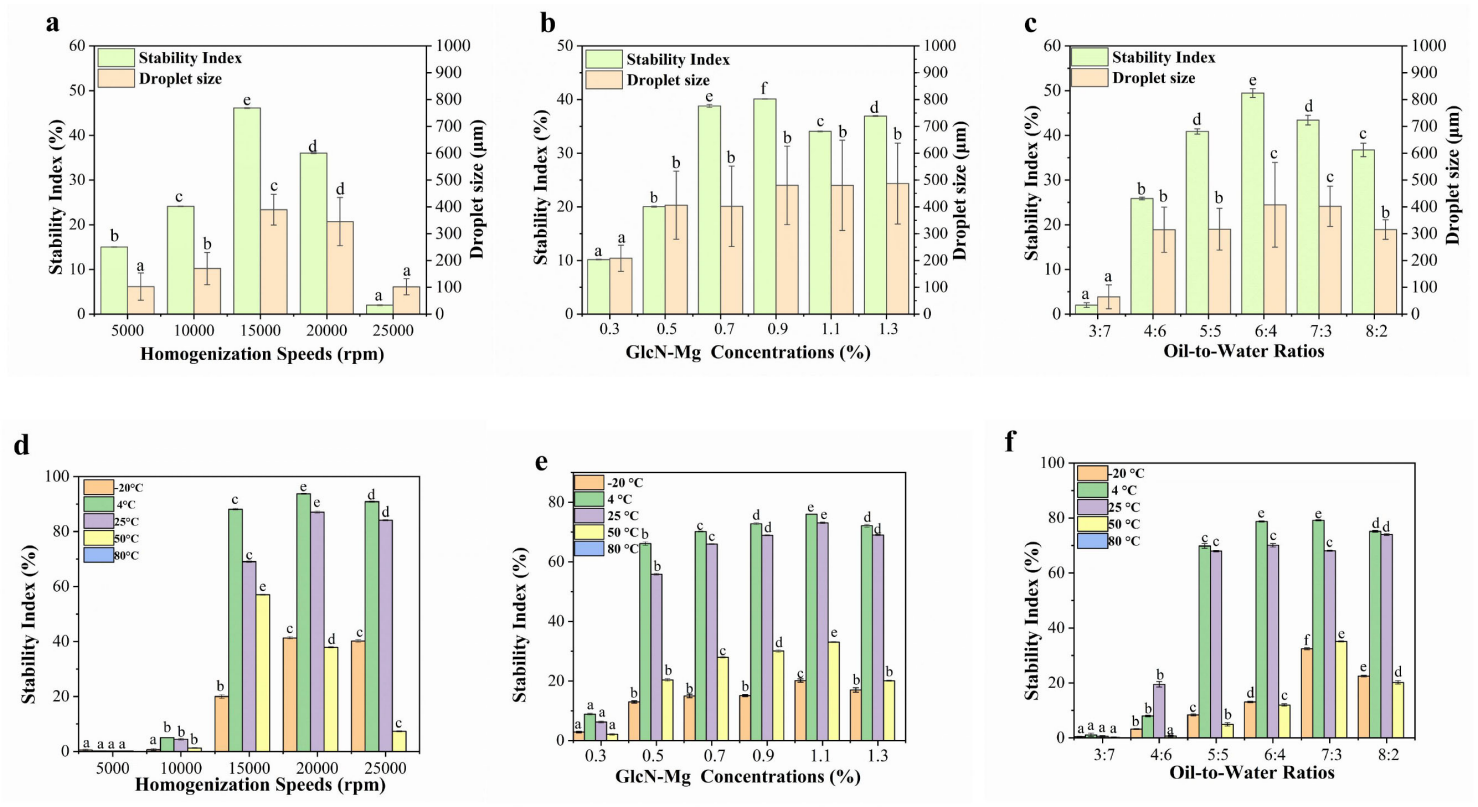
**(a)** Droplet size and centrifugal stability of glucosamine-magnesium composite stabilized Pickering emulsion (GlcN-Mg PE) with different homogenization speeds. **(b)** Droplet size and centrifugal stability of GlcN-Mg PE with different glucosamine-magnesium composite (GlcN-Mg) concentrations. **(c)** Droplet size and centrifugal stability of GlcN-Mg PE with different oil-to-water ratios. **(d)** Temperature stability of GlcN-Mg PE with different homogenization speeds. **(e)** Temperature stability of GlcN-Mg PE with different GlcN-Mg concentrations. **(f)** Temperature stability of GlcN-Mg PE with different oil-to-water ratios.

The droplet size and centrifugal stability of GlcN-Mg PE are shown in Figures 4a–c. Centrifugation served as a rapid method to assess emulsion stability (56). Larger droplet sizes were correlated with enhanced centrifugal stability of GlcN-Mg PE. Under the influence of different homogenization speeds, droplet sizes of GlcN-Mg PE varied from 102.14 ± 29.95 to 389.34 ± 57.25 μm. Extremely high homogenization speeds induced droplet disintegration via shear forces, destabilizing the emulsion system (57). This observation aligned with centrifugal stability trends: below a critical threshold, the SI increased with homogenization speed, attaining the maximum value of 46.10 ± 0.07% at 15,000 rpm. However, speeds exceeding 20,000 rpm induced interfacial detachment. When the speed increased to 25,000 rpm, the SI dropped to 2.00 ± 0.07%. Moreover, the droplet size of GlcN-Mg PE increased with increasing GlcN-Mg concentration. Among the tested concentrations, the largest droplet size was observed in GlcN-Mg PE with 0.9% GlcN-Mg concentration, corresponding to optimal stability. Within the 0.3%–0.9% range, the SI increased from 10.17 ± 0.11% to 40.10 ± 0.07%. Beyond 0.9% (w/v) GlcN-Mg concentration, the SI did not rise any further despite continued GlcN-Mg addition. This was attributed to repulsive forces between particles that induced structural defects (58). As the oil-to-water ratio increased from 3:7 to 6:4, droplet size increased from 64.67 ± 44.49 to 407.70 ± 157.89 μm, likely due to enhanced droplet packing. This correlated with SI increase from 2.01 ± 0.53% to 49.47 ± 0.98%. However, excessive oil content reduced droplet size to 315.38 ± 36.34 μm and SI to 36.73 ± 1.51%, attributed to limitations in emulsifier redistribution during phase transformation.

#### 3.8.2 Temperature stability

The temperature stability of GlcN-Mg PE was shown in Figures 4d–f. GlcN-Mg PE exhibited temperature-dependent stability profiles. Freezing at −20 °C induced water crystallization, which mechanically compressed oil droplets. It triggered irreversible separation after thawing (59–62). GlcN-Mg PE maintained structural integrity between 4 °C and 25 °C but degraded above 50 °C, with complete phase separation observed at 80 °C (SI = 0%). This thermal degradation was attributed to weakened GlcN-Mg interfacial adsorption and disintegration of the composite network, driven by intensified Brownian motion (63). The SI remains stable initially but decreases progressively at higher temperatures, indicating that excessive heating led to thermal denaturation and aggregation of GlcN-Mg adsorbed on the droplet surface. The temperature stability of GlcN-Mg PE with different homogenization speeds followed a discernible trend. When prepared at low homogenization speeds of 5,000–10,000 rpm, the SI of GlcN-Mg PE was less than 10%. GlcN-Mg PE processed at low speeds exhibited insufficient thermal resistance due to incomplete interfacial coverage. Optimal performance was achieved at 20,000 rpm, with SI values reaching 93.73 ± 0.18% at 4 °C and 87.07 ± 0.42% at 25 °C. When the homogenization speed increased to 25,000 rpm, the SI dropped slightly to 84.10 ± 0.20% at 25 °C. Compared with other concentrations, GlcN-Mg PE with 1.10% GlcN-Mg concentrations exhibited the highest temperature stability. Increasing the concentration from 0.3% to 1.3% initially enhanced stability, followed by a slight decrease at higher concentrations. GlcN-Mg PE with different oil-to-water ratios showed similar variation patterns as above. At an oil-to-water ratio of 3:7, the SI was below 5%. At 4 °C, the SI for oil-to-water ratios of 6:4 and 7:3 was 78.73 ± 0.22% and 79.17 ± 0.22%, respectively. Optimal thermal resistance emerged at intermediate ratios of 6:4 and 7:3, facilitated by densely packed droplet architectures.

### 3.9 GlcN-Mg PE *in vitro* simulated gastrointestinal digestion

**FIGURE 5:**
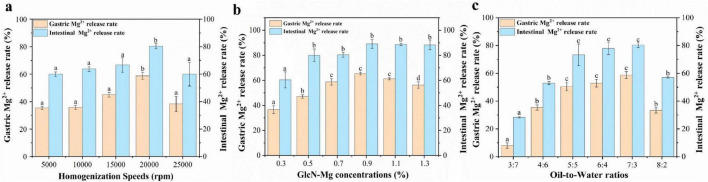
**(a)** Gastrointestinal digestibility of of glucosamine-magnesium composite stabilized Pickering emulsion (GlcN-Mg PE) with different homogenization speeds. **(b)** Gastrointestinal digestibility of GlcN-Mg PE with different GlcN-Mg concentrations. **(c)** Gastrointestinal digestibility of GlcN-Mg PE with different oil-to-water ratios.

The performance of GlcN-Mg PE in simulated gastrointestinal digestion is illustrated in Figures 5a–c. All samples exhibited an initial rapid release of Mg^2+^, followed by a slower release rate, eventually reaching a plateau. The GlcN-Mg PE demonstrated controlled Mg^2+^ release throughout the *in vitro* simulated gastrointestinal digestion, reaching 80.42 ± 1.94% at the endpoint of intestinal phase. The maximum Mg^2+^ release occurred under the conditions of a homogenization speed of 20,000 rpm, a GlcN-Mg concentration of 0.90%, and an oil-to-water ratio of 7:3. Compared with the conditions under which the optimal Mg^2+^ release rate occurred, excessive homogenization caused the collapse of the emulsion structure; higher GlcN-Mg concentrations led to defects at the oil-to-water interface, and excessively high oil content led to phase transformation. These scenarios hindered the interaction with acid and restricted the entry of enzymes, thereby reducing Mg^2+^ release. After gastric digestion, the Mg^2+^ release rate was 65.42 ± 1.11%. During the gastric phase, acidic hydrolysis protonated amino/hydroxyl groups within GlcN-Mg coordination bonds, neutralizing electrostatic repulsion between composite particles (64). This triggered rapid aggregation of composites, thereby accelerating Mg^2+^ diffusion into gastric fluid. Under these conditions, steric hindrance provided by the GlcN-Mg composite became the primary factor governing GlcN-Mg PE behavior. The extent of gastric release depended on acid accessibility at the interface. Upon exposure to simulated intestinal fluid, the Mg^2+^ release rates of GlcN-Mg PE increased again, reaching 80.42 ± 1.94% at the end of digestion. Lipase-mediated cleavage of ester bonds generated amphiphilic metabolites, such as free fatty acids (FFA) and monoacylglycerols (MAG) (65, 66). These surface-active compounds competitively displaced GlcN-Mg from oil-to-water interfaces, forming mixed micelles that solubilized Mg^2+^-loaded droplets (67–72). Lipid substrates moderately maintained droplet stability during gastric transit while enabling disintegration in the intestine.

## 4 Conclusion

This study developed a GlcN-Mg composite as a novel Pickering emulsion stabilizer. Structural analysis revealed that Mg^2+^ coordination significantly reduced the particle size of GlcN⋅HCl from 1,117 ± 222.58 to 393.8 ± 45.42 nm, expanded its crystal lattice, and created a porous structure with a 6.1 ± 0.30 m^2^/g surface area and a 22.9 ± 1.80 nm average pore size, thereby facilitating interfacial anchoring in Pickering emulsions. GlcN-Mg demonstrates robust stability in food matrices under critical stressors, while exhibiting controlled Mg^2+^ release with low gastric release (26.11 ± 0.44% at 120 min) and high intestinal bioavailability (86.11 ± 0.67% at 240 min). Microstructural analysis showed that GlcN-Mg PE exhibited a W/O structure, with a three-dimensional network surrounding the emulsion droplets. Rheological analysis revealed non-linear viscosity changes, peaking at 176.04 ± 0.64 mPa⋅s. The preparation conditions for the optimal stability were a homogenization speed of 20,000 rpm, a GlcN-Mg concentration of 0.9%, and an oil-to-water ratio of 7:3. GlcN-Mg PE prepared under the best conditions maintained higher stability under three environmental stimuli: a temperature stability of 93.73 ± 0.18% at 4 °C, a centrifugal stability of 43.40 ± 1.07%. Experiments showed that using excessively high homogenization speeds, adding excessive GlcN-Mg, or using an imbalanced oil content all damaged the emulsion structure and reduced stability. During simulated digestion, GlcN-Mg PE demonstrated a controlled Mg^2+^ release rate of 65.42 ± 1.11% in gastric phase and 80.42 ± 1.94% in intestinal. Though slightly lower than those observed with the GlcN-Mg composite, it demonstrated effective sustained release throughout digestion process. The stability and controlled release properties of GlcN-Mg PE observed *in vitro* suggest potential for further exploration in functional food or nutritional supplement contexts. These findings suggest that GlcN-Mg has potential as a stabilizer for stimuli-responsive systems under the tested conditions. This work connects coordination chemistry with emulsion science, providing insights that may inform the design of adaptive colloids in food and pharmaceutical technologies.

## Data Availability

The original contributions presented in this study are included in this article/supplementary material, further inquiries can be directed to the corresponding author.
